# Testing low dietary crude protein and high fat levels as a strategy to mitigate heat stress in broilers

**DOI:** 10.1186/s40104-025-01297-4

**Published:** 2025-12-19

**Authors:** Renée De Baets, Sofie Van Nerom, Kobe Buyse, Gunther Antonissen, Jeroen Degroote, Evelyne Delezie

**Affiliations:** 1 Animal Science Unit, Flanders Research Institute for Agriculture, Fisheries and Food, Merelbeke-Melle, 9090 Belgium; 2 Department of Pathobiology, Pharmacology and Zoological Medicine, Faculty of Veterinary Medicine, Ghent University, Merelbeke-Melle, 9820 Belgium; 3 Department of Veterinary and Biosciences, Faculty of Veterinary Medicine, Ghent University, Merelbeke-Melle, 9820 Belgium; 4 Laboratory for Animal Nutrition and Animal Product Quality, Department of Animal Sciences and Aquatic Ecology, Faculty of Bioscience Engineering, Ghent University, Ghent, 9000 Belgium

**Keywords:** Broiler, Crude fat, Crude protein, Energy, Heat stress

## Abstract

**Background:**

Fast-growing broilers are poorly adapted to heat. Adjusting feed composition may mitigate heat stress (HS) effects in temperate climates, while maintaining performance and health during cooler days.

**Methods:**

One thousand nine hundred and twenty Ross 308 male broilers were housed in 64 pens in 4 climate-controlled rooms, 2 under cyclical HS (d 28–43; 32 ± 2 °C; 60%–70% RH; 09:30–15:30) and 2 under thermoneutral (TN) conditions. In the finisher phase, broilers were allocated to 4 dietary treatments, analyzed values are given except for metabolizable energy (ME): low crude protein (CP) and control fat (LowCP-ConF; 17.0% CP, 5.9% crude fat (CF), 2,925 kcal/kg ME), low CP and high fat (LowCP-HighF; 17.2% CP, 7.9% CF, 3,019 kcal/kg ME), control CP and high fat (ConCP-HighF; 18.1% CP, 8.0% CF, 2,992 kcal/kg ME) and a basal control (ConCP-ConF; 18.7% CP, 6.3% CF, 2,913 kcal/kg ME). LowCP diets contained control levels of digestible amino acids.

**Results:**

During the finisher phase, compared to control CP levels, LowCP increased average daily feed intake (ADFI) (+ 2.15%; *P* = 0.020) and affected average daily gain (ADG) and feed conversion ratio (FCR) negatively under TN (−3.77% and +6.49%; *P* = 0.003 and *P* < 0.001, respectively), but not during HS. Compared to control CF, HighF decreased ADFI during TN and HS (−3.16% and −3.17%; *P* < 0.001 and *P* = 0.022) and reduced ADG in TN groups (−3.17%; *P* = 0.010), but not during HS. Mortality was higher in broilers receiving HighF during HS (*P* = 0.040). Slaughter weights were unaffected. LowCP decreased plasma uric acid and lactate dehydrogenase levels during TN, but increased plasma glucose during HS. LowCP increased breast meat redness (a*) during TN and HS (*P* < 0.05). HighF decreased fat (−1.68%; *P* = 0.017), but increased protein levels (+1.53%; *P* < 0.001) in breast meat of HS-broilers.

**Conclusion:**

LowCP and HighF impaired performance under TN but not under HS. HighF increased mortality under HS, yet improved breast meat composition. These findings highlight the challenge of designing an optimal diet for both conditions and underscore the need to better understand amino acid needs and energy-to-protein ratios during HS.

## Introduction

Heat waves during summer are becoming more frequent in temperate climates, intensifying the concerns regarding the impact of heat stress (HS) effects on livestock [1]. High-productive livestock in these regions can be sensitive and poorly adapted to unexpected temperature increases [2, 3]. Broilers, in particular, have a fast metabolism and highly efficient growth rate, rendering them susceptible to HS. According to Thornton et al. [4], broilers may experience HS when the temperature-humidity index (THI), calculated using the formula of Thom et al. [5], exceeds 74. HS hampers animal welfare and health [6], leading also to substantial production losses for farmers. While prevention of HS is essential, adjusting the feed composition during summer can also be beneficial to alleviate the impact of HS [7]. However, studies on this topic are limited, often conducted under high continuous temperatures and therefore not fully representative of temperate climates with cyclical HS. Additionally, there is ongoing debate regarding the optimal nutritional strategy for modern fast-growing broiler strains under HS, particularly whether crude protein and dietary fat levels should be reduced or increased [8].

Protein has a lower energy efficiency than fat and carbohydrates due to the energy required for nitrogen excretion [9]. The net energy-to-metabolizable energy (NE/MEn) ratios of digestible carbohydrates, fat, and proteins are approximately 68%, 86% and 76%, respectively. However, the true heat increment of protein should be higher because of the energy costs associated with nitrogen retention during deamination, resulting in a net energy-to-metabolizable energy ratio of only 59% [10]. As a result, diets high in crude protein (CP) can increase heat production, which may affect performance negatively during HS [11, 12]. Reducing CP levels has been suggested as a strategy to lower diet-induced thermogenesis [8, 9, 13]. Additionally, a lower CP diet reduces nitrogen emissions, making it both cost-effective and environmentally beneficial [14–16]. Although it is important to note that reducing CP without supplementing crystalline amino acids to meet the protein requirement or failing to balance the amino acid profile can lead to amino acid deficiencies and decreased performance. As a result, broilers may increase their feed intake to compensate for these deficiencies, paradoxically increasing their overall energy intake and heat production [17–21]. Therefore, maintaining an appropriate amino acid balance is essential when reducing protein [8].

Because HS reduces feed intake, while also increasing energy requirements, increasing dietary energy has been proposed as a compensatory strategy to maintain growth performance [7, 22]. Fat supplementation may be particularly beneficial, as fat generates a lower heat increment compared to protein and carbohydrates, thereby reducing heat production [23, 24]. Added fat also slows the intestinal transit down, enhancing nutrient absorption and digestibility [6, 25]. Providing energy via the increased inclusion of dietary fat while also reducing the heat increment via a lower CP level may therefore provide advantages under HS conditions. For example, Zaman et al. [26] observed a higher body weight gain when broilers received a diet with low CP combined with high energy in a hot climate. Although careful attention should be given to the ratios between macronutrients (protein, lipid and carbohydrate), as these ratios may affect the outcomes of dietary adjustments differently, highlighting the importance of studying their interactions [27]. Furthermore, the type of fat included in the diet is important, as high levels of polyunsaturated fats may increase oxidative stress under HS conditions [28].

Implementing a specialized seasonal diet in common practice could come with a few practical considerations and constraints. For example, because it is difficult to reliably predict upcoming heat waves for a large time window in a temperate climate, it is essential that a summer diet both alleviates HS effects as well as supports optimal performance and animal health during TN conditions. Therefore, the present study tested high dietary fat and low crude protein levels separately, but also combined, compared to control levels under both TN and cyclical HS conditions to assess their HS mitigating effects without compromising overall performance.

## Materials and methods

All experimental procedures in this study complied with the European guidelines for the care and use of animals in research (Directive 2010/63/EU) and were approved by the Ethical Committee of the Research Institute for Agriculture, Fisheries and Food (ILVO), Merelbeke-Melle, Belgium under authorization number 2022/214.

### Birds and experimental design

A total of 1,920 Ross 308 day-old male broilers were obtained from a commercial hatchery (Belgabroed, Merksplas, Belgium). The birds were housed in the poultry experimental facility and were randomly allocated to 64 pens in 4 climate-controlled rooms (16 pens/room). Each pen, with dimensions measuring 2.45 m × 0.8 m, housed 30 broilers. Broilers were housed on a solid floor with wood shavings (3 kg/m^2^) and were kept in 18L6D schedule from d 6 to 42. The last day and first week a 23L1D cycle was used. Broilers were reared using a 3-phase diet scheme with a starter (d 0 to 11), grower (d 11 to 25), and finisher (d 25 to 42) diet. The starter diet was a mash feed, while the grower and finisher diets were pelleted. Feed and drinking water were available ad libitum. Dietary treatments were applied only during the finisher phase to test their effects specifically during HS episodes, which were induced only during this period. All pens were allocated to one out of 4 dietary treatments according to an alternate block design resulting in 8 treatment groups: 4 dietary × 2 climate treatments. Due to the limited number of replicates per climate treatment (2 rooms per climate condition), statistical analysis focused solely on the interaction between dietary crude protein and fat levels (CP × CF) within each thermal environment separately. The nutrient composition, calculated and analyzed, of all diets is shown in Table 1. A basal finisher diet was formulated to meet the birds’ requirements with control levels of crude protein (CP), crude fat (CF) and metabolizable energy (AME_n_) (ConCP-ConF; calculated values: 18.2% CP, 8.0% CF, 2,913 kcal/kg ME). In the second diet, a lower crude protein level was introduced (LowCP-ConF; calculated values: 17.3% CP, 7.8% CF, 2,925 kcal/kg ME) by reducing soybean meal with circa 2.78% and by increasing the amount of wheat with 2.85%. The digestible amino acid levels and the crude fat level remained similar. The third diet similarly contained a low crude protein level by lowering soybean meal with 2.21% while the crude fat level was increased by additionally including 0.5% of soy oil and 1.21% of animal fat (LowCP-HighF; calculated values: 17.3% CP, 9.7% CF, 3,019 kcal/kg ME). The last diet only got higher crude fat by including 0.5% of soy oil and 1.21% animal fat (ConCP-HighF; calculated values: 18.2% CP, 9.6% CF, 2,992 kcal/kg ME). All diets contained the same ingredients and were supplemented with equal amounts of non-starch polysaccharide enzymes (100 mg/kg, Ronozyme Multigrain, DSM, Heerlen, the Netherlands), phytase (1,000 mg/kg, Ronozyme HiPhos, DSM, Heerlen, Netherlands), and diclazuril (500 mg/kg, Coxiril, Huvepharma, Sofia, Bulgaria). Diets with the same level of fat were kept isoenergetic.

**Table 1:** Feed composition of the diets

Item	**Starter (d 0**–**11)**	**Grower (d 11**–**25)**	**Finisher (d 25**–**42)** **LowCP-ConF**	**Finisher (d 25**–**42)** **LowCP-HighF**	**Finisher (d 25**–**42)** **ConCP-HighF**	**Finisher (d 25**–**42)** **ConCP-ConF**
Ingredient, %						
Wheat	63.42	65.24	69.61	67.00	64.63	66.76
Corn	5.00	5.00	5.00	5.00	5.00	5.00
Soybean	3.00	4.00	4.00	4.00	3.85	4.00
Soybean meal (48% crude protein)	22.47	18.79	14.33	14.90	17.69	17.11
Soy oil	1.00	1.00	0.85	1.50	1.50	1.00
Animal fat	1.00	2.22	2.69	4.00	4.00	2.79
Mineral and vitamin premix^1^	1.00	1.00	1.00	1.00	1.00	1.00
Bi-calcium phosphate	0.921	0.612	0.485	0.655	0.508	0.504
Sodium chloride	0.160	0.168	0.120	0.120	0.147	0.143
Sodium-bicarbonate	0.281	0.233	0.298	0.297	0.224	0.231
Limestone	0.680	0.765	0.586	0.479	0.552	0.556
DL-Methionine	0.314	0.275	0.264	0.278	0.253	0.251
L-Lysine-HCl	0.386	0.365	0.411	0.413	0.329	0.338
L-Threonine	0.205	0.176	0.193	0.198	0.160	0.160
Coccidiostaticum	0.050	0.050	0.050	0.050	0.050	0.050
NSP enzyme	0.010	0.010	0.010	0.010	0.010	0.010
Phytase	0.100	0.100	0.100	0.100	0.100	0.100
Calculated nutrient composition, %						
Dry matter	88.49	88.70	87.78	88.04	87.97	87.77
Crude protein	20.50	19.20	17.3	17.3	18.2	18.2
Crude fat	6.04	7.47	7.80	9.66	9.60	8.00
Crude fiber	3.57	3.56	3.69	3.60	3.59	3.66
Crude ash	4.75	4.47	4.01	4.02	4.06	4.07
Non-starch polysaccharides	12.20	11.87	10.94	10.80	11.05	11.17
Metabolizable energy, kcal/kg	2,768	2,868	2,925	3,019	2,992	2,913
SID Lysine	1.15	1.06	0.97	0.98	0.98	0.98
SID Methionine + cysteine	0.86	0.80	0.74	0.75	0.75	0.75
SID Threonine	0.78	0.71	0.66	0.67	0.67	0.67
SID Valine	0.77	0.72	0.64	0.64	0.69	0.69
SID Isoleucine	0.71	0.65	0.58	0.58	0.63	0.62
SID Tryptophane	0.22	0.20	0.18	0.18	0.19	0.19
SID Arginine	1.13	1.04	0.90	0.90	0.98	0.97
Calcium	0.85	0.80	0.70	0.70	0.70	0.70
Available P	0.40	0.35	0.31	0.33	0.32	0.32
NaCl + KCl, mEq/kg	223	204	190	190	198	200
Lineolic acid (18:2), %	0.25	0.28	0.27	0.33	0.33	0.29
Analyzed nutrient composition, %^2^						
Gross energy^3^, kcal/kg			4,051	4,187	4,206	4,159
Crude protein^4^	20.04	17.77	16.96	17.15	18.06	18.67
Crude fat^5^	4.42	5.54	5.85	7.87	8.01	6.32
Crude ash^6^	5.33	4.43	4.12	4.22	4.56	4.71
Crude fiber^7^	2.98	2.74	2.46	2.55	2.53	2.55

*LowCP-ConF *Low crude protein with control fat, *LowCP-HighF *Low crude protein with high fat, *ConCP-HighF *Control crude protein with high fat, *ConCP-ConF *Control crude protein with control fat

^1^Mineral and vitamin premix composed of vitamin A/retinyl acetate 3a672a (1,000,000 IU/kg); vitamin D_3_ E671 (299,999.4 IU/kg); vitamin E 3a700 (all-rac-alpha-tocopheryl acetate) (5,000 IU/kg); vitamin K_3_ 3a710 (250 mg/kg); vitamin B_1_/thiamine mononitrate 3a821 (200 mg/kg); vitamin B_2_/riboflavin (500 mg/kg); calcium D-pantothenate 3a841 (1,500 mg/kg); vitamin B_6_/pyridoxine hydrochloride 3a831 (400 mg/kg); vitamin B_12_/cyanocobalamine (2.5 mg/kg); niacinamide 3a315 (3,000 mg/kg); folic acid 3a316 (100 mg/kg); biotin/D-(+)-biotin 3a880 (15 mg/kg); choline chloride 3a890 (68,965.5 mg/kg); iron (II) sulphate (monohydrate)—iron E1 (4,920 mg/kg); copper (II) sulphate (pentahydrate)—copper E4 (2,000 mg/kg); zinc oxide 3b603 (6,000 mg/kg); manganese (II) oxide—manganese E5 (9,590.2 mg/kg); calcium iodate (anhydrous)—iodine 3b202 (120 mg/kg); sodium selenite—selenium E8 (36 mg/kg); sepiolite E562 (700 mg/kg); propyl gallate E310 (200 mg/kg); BHT E321 (300 mg/kg); citric acid E330

^2^Fresh weight basis

^3^Based on ISO [29] method 9831

^4^Crude protein (CP) (N × 6.25) based on ISO [30] method 5983-2

^5^Crude fat-B (CF) based on ISO [31] method 6492

^6^Based on ISO [32] method 5984

^7^Based on American Oil Chemists' Society (AOSC) [33]

Afterwards, feed was analyzed for metabolizable energy [29], crude protein (N × 6.25) [30], crude fat-B (hydrolysis with HCl followed by extraction with petroleum ether) [31], crude ash [32], and crude fiber [33].

**Fig. 1:**
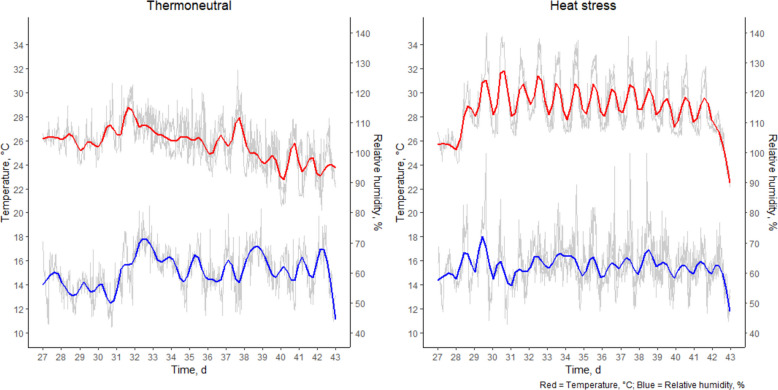
Stable climate from d 28 till d 42. Temperature (°C; red, upper line) and relative humidity (%; blue, lower line)

In the first week, the poultry house temperature was 32 °C. Afterwards, temperature was gradually decreased by 4 °C each week until 22 °C. Starting from d 28, half of the animals (32 pens; 2 rooms) were subjected to cyclic HS until slaughter age (d 42). The other half was kept at TN conditions, i.e. a constant room temperature at 22 °C. In HS compartments, temperature was increased to 32 ± 2 °C with a RH of 60% to 70% for 6 h (09:30–15:30) (Fig. 1). During the rest of the day, temperature was maintained around 26 ± 1 °C with a RH of 55% to 65% to imitate a natural heat wave. Heating was turned off, and ventilation was increased gradually to allow the room temperature to decrease gently, thus not exposing the animals to a sudden temperature change. Ambient temperature and RH were continuously measured in each room at two positions, i.e. in the middle of the room and the other one inside the chicken pen, at a height of 1 m and 50 cm respectively. The temperature-humidity index (THI) was calculated according to the formula of Thom [5]: THI = 0.8 × T + [(RH/100) × (T – 14.3)] + 46.4; where T represents temperature in °C and RH represents relative humidity as a percentage.

### Measurements

#### Animal performance

Animal live body weight (BW), feed offered and feed leftovers were recorded at d 0, 11, 25, and 38 at the pen level to assess average daily gain (ADG; g/d), average daily feed intake (ADFI; g/d), and feed conversion ratio (FCR). Mortality and culled birds were registered daily to correct performance indices using the number of animal days in the trial.

#### Body temperature, breathing frequency, and panting percentage

At d 28, 30, 32, 35, and 37, body temperature (Tb) and breathing frequency were measured in two chickens per pen, 4 h after the onset of HS, to ensure that potential effects on physiological parameters could be detected, as demonstrated by Lin et al. [34]. On d 25, all birds were weighed and two birds with an average weight were selected and individually color-marked. From these, one bird per pen was consistently used for repeated measurements of cloacal temperature using a digital thermometer (Digi-Vet SC 12, Kruuse, Langeskov, Denmark). Video recordings of each pen were made to determine breathing frequency. Afterwards, a human annotator analyzed the videos to determine the breathing frequency of the two marked chickens per pen by counting the number of breaths in a 30 s episode. Additionally, each pen was given a panting score (%) by counting the number of birds that were panting compared to the total number of birds in that pen. At d 25 and 38, litter was visually scored for its consistency using a scoring system. At the same moment, hock and footpad lesions were scored in 8 randomly selected birds per pen. All scoring systems were based on the Welfare quality assessment protocols [35].

#### Sample collection and processing

Sampling started 4 h after the onset of HS to ensure HS effects and sampling was spread over two days to keep temperature differences to a minimum. At both d 38 and 39, one marked chicken per pen was sampled. Live BW and body temperature were noted before euthanasia by intravenous injection with sodium pentobarbital 20% (Kela, Hoogstraten, Belgium). Blood samples were collected by exsanguination in serum tubes (4 mL; no anticoagulant) and EDTA tubes (8 mL). Serum samples were used to measure aspartate transaminase (AST; IU/L), lactate dehydrogenase (LDH; IU/L), creatine kinase (CK; IU/L) and uric acid (mg/dL) (DGZ, Torhout, Belgium). The EDTA samples were centrifuged (1,500 × *g*, 10 min, 4 °C) and plasma aliquots (2 mL) were made in Eppendorf tubes and frozen at −80 °C until malondialdehyde (MDA) analysis according to the method of Grotto et al. [36] with slight modifications [37] as described in De Baets et al. [38]. Glucose (mg/dL) was measured using a commercial glucose meter (OneTouch Verio Reflect, LifeScan Belgium, Westerlo, Belgium). Similarly, lactate (mmol/L) was determined using a lactate analyzer (The EDGE Lactate Analyzer, RDSM, Hasselt, Belgium). Abdominal fat was dissected and weighed. The liver was removed and color (Hunterlab Miniscan 45/0, AMETEK Hunterlab, Reston, VA) was measured with CIE L*a*b*, where L*, a* and b* values represent lightness, red/green and blue/yellow respectively as axes on a 3-dimensional color scheme. A liver sample of the right lobe was collected, flash frozen in liquid nitrogen and stored at −80 °C for further oxidative analysis. Lastly, the right pectoralis major was dissected, weighed and stored at −20 °C for further analysis. Breast muscles were lyophilized and analyzed on dry matter basis [39]: lipids were measured based on ISO method 6492, and crude protein was measured based on ISO method 5938−2 [30, 31].

#### Slaughter yield and meat quality

At the end of the experiment, 31 birds per treatment were selected ad random, individually marked, weighed, and fasted overnight until transport to a commercial slaughterhouse. The eviscerated carcasses were retrieved and stored at 4 °C until the following day. Carcass yield was determined as eviscerated carcass weight relative to live weight before slaughtering. All carcasses were cut into different parts to determine carcass, wing, leg (thigh and drumstick) and breast meat yield as their weight relative to eviscerated carcass weight. All parts were skin-on and bone-in, except for the breast. Breast fillets were scored for abnormalities (wooden breast, white striping and spaghetti meat) by a trained professional. The different meat quality parameters were determined using the breast meat (pectoralis major muscles) and included the following measurements in chronological order: temperature (°C), pH, color, thawing loss (%), cooking loss (%) and shear force (N) was measured as described by Buyse et al. [40].

### Statistical analysis

Statistical analysis was performed with R version 4.1.2 for Windows [41]. Interaction effects between diet and climate conditions were not included in the statistical model, as the available replication (2 rooms per climate condition) did not allow for reliable estimation of three-way or two-way interactions involving climate. Statistics were performed separately on the TN and HS data. The pen was considered the experimental unit. For performance, linear mixed models were made with interaction effects of ‘crude protein’ and ‘crude fat’ as fixed factors and ‘block’ as random factor. The ‘block’ variable encompassed four neighboring pens, each containing all four treatments. For meat quality analysis and physiology parameters, a linear mixed model was used with interaction effects of ‘crude protein’ and ‘crude fat’ as fixed factors and ‘block’ and ‘pen’ as random factor. If absolute weights were used, bodyweight was used as covariable in the model. For breathing frequency and body temperature, a linear mixed model was used with ‘measurement day’ and interaction effects of ‘crude protein’ and ‘crude fat’ as fixed factors and ‘block’ and ‘pen’ as random factor. For panting percentage, a generalized mixed model was used with ‘measurement day’ and interaction effects of ‘crude protein’ and ‘crude fat’ as fixed factors and ‘block’ and ‘pen’ as random factor. Mortality was analyzed using logistic regression (generalized linear model with binomial distribution), with ‘crude protein’ and ‘crude fat’ as fixed factors. For lesion and breast meat scores, a cumulative link model was used with ‘crude protein’ and ‘crude fat’ as fixed effects. In all statistical models mentioned above, if the interaction was not significant, the interaction term was removed from the model and the main effects were tested. Linear model assumptions (normality and homoscedasticity) were verified by a visual check of the residuals plots (q-q plots and histograms). Tukey’s range test (Honest Significant Difference, HSD) was used to obtain adjusted *P*-values to account for multiple comparisons, with level of significance α = 0.05. Tables and figures report the mean ± standard error of the mean (SEM). In the Results section, numerical values are presented as mean ± standard deviation.

## Results

### Nutrient composition

All dietary treatments were analyzed and compared to the calculated nutrient composition. Analyzed crude protein content in the grower diet was lower than calculated (17.77% vs. 19.20%). Crude protein content of the four finisher diets was also relatively lower, but a reduced crude protein level in LowCP-ConF (16.96%) and LowCP-HighF (17.15%) was still obtained in relation to ConCP-ConF (18.67%) and ConCP-HighF (18.06%). The same was observed in crude fat: analyzed values were lower in all treatments, but the level of fat in LowCP-HighF (7.87%) and ConCP-HighF (8.01%) was still substantially higher than ConCP-ConF (6.32%) and LowCP-ConF (5.85%). Analyzed gross energy content was slightly higher in LowCP-HighF (4,187 kcal/kg) and ConCP-HighF (4,206 kcal/kg) compared to LowCP-ConF (4,051 kcal/kg) and ConCP-ConF (4,159 kcal/kg).

### Climate conditions

The climate of the four different rooms is shown in Fig. 1. During the HS period (09:30–15:30), the average temperature in both heat stress rooms was 31.8 ± 0.67 °C and 32.4 ± 0.53 °C, with a RH of 63.9% ± 0.44% and 71.6% ± 2.89% respectively. This resulted in a THI of 83.0 ± 1.04 and 85.2 ± 1.32 high to extreme HS [4]. During the TN period (15:30–09:30 h), the temperature in these rooms averaged 28.2 ± 0.32 °C and 28.7 ± 0.28 °C, with a RH of 59.6% ± 1.84% and 63.6% ± 1.82%, respectively. The TN rooms, which remained TN throughout the trial, had an average temperature of 26.8 ± 1.55 °C and 26.0 ± 1.54 °C, with a RH of 63.8% ± 6.98% and 63.2% ± 6.53%, respectively.

### Animal performance

There were no interaction effects between crude protein and fat for none of the performance parameters. Pre-experimental performance indices were included in the table to confirm the absence of baseline differences between treatment groups (Table 2). During both TN and HS conditions, there were no significant effects of the diet treatments on BW. However, broilers fed the LowCP diet had a significantly higher ADFI (185.7 ± 6.42 g/d) during d 25–38 compared to control levels (181.8 ± 6.61 g/d) under TN conditions (*P* = 0.020). Additionally, LowCP diets also significantly reduced ADG (94.4 ± 3.41 g/d) compared to diets with control levels (98.1 ± 3.99 g/d) under TN conditions (*P* = 0.003). Consequently, the FCR during this period was higher in the treatments fed the LowCP diet (1.97 ± 0.036) compared to treatments with control levels (1.85 ± 0.054) (*P* < 0.001). This effect was also significant considering the total rearing period (*P* < 0.001). Under HS conditions, ADFI, ADG and FCR were not significantly different when considering CP content, regardless of CF content.

**Table 2:** Broiler performance during starter, grower and finisher with treatments applied during finisher under TN and HS conditions

Item	**TN**								**HS**							
	**LowCP-ConF**	**LowCP-HighF**	**ConCP-HighF**	**ConCP-ConF**	**SEM**	***P*-value**			**LowCP-ConF**	**LowCP-HighF**	**ConCP-HighF**	**ConCP-ConF**	**SEM**	***P*-value**		
						**CP × CF**	**CP**	**CF**						**CP × CF**	**CP**	**CF**
BW d 0	44.2	44.4	44.9	44.8	0.26				43.9	44.5	43.8	43.7	0.28			
BW d 11	262.8	265.3	262.7	271.6	6.05				279.6	278.4	274.5	285.6	8.89			
BW d 25	1,172.8	1,179.9	1,139.5	1,169.1	27.8				1,173.7	1,181.5	1,164.0	1,205.4	26.9			
BW d 38	2,423	2,385	2,398	2,463	38.7	0.625	0.327	0.605	2,202	2,187	2,178	2,262	35.4	0.264	0.408	0.113
ADFI d 0–11	25.0	25.4	25.4	25.4	0.36				26.0	25.6	25.6	25.6	0.52			
ADFI d 11–25	87.1	87.7	84.7	87.4	1.79				89.8	89.7	87.8	91.1	2.03			
ADFI d 25–38	188.4	182.9	178.7	184.9	2.11	0.838	**0.020**	**< 0.001**	164.4	159.5	158.6	161.4	2.24	0.508	0.227	**0.022**
ADFI d 0–38	101.8	100.3	97.8	100.8	1.34	0.405	0.074	**0.021**	95.0	93.2	92.3	94.4	1.36	0.874	0.415	0.057
ADG d 0–11	18.2	18.4	18.2	18.9	0.50				19.6	19.5	19.2	20.2	0.74			
ADG d 11–25	65.0	65.3	62.6	64.1	1.66				63.9	64.5	63.5	65.7	1.39			
ADG d 25–38	96.2	92.7	96.8	99.5	1.22	0.730	**0.003**	**0.010**	79.1	77.3	78.0	81.3	1.73	0.637	0.361	0.108
ADG d 0–38	61.0	60.0	60.3	62.0	0.99	0.626	0.335	0.059	55.3	54.9	54.7	56.9	0.91	0.267	0.398	0.110
FCR d 0–11	1.37	1.39	1.40	1.35	0.03				1.34	1.32	1.34	1.27	0.04			
FCR d 11–25	1.34	1.34	1.36	1.37	0.01				1.41	1.39	1.38	1.39	0.01			
FCR d 25–38	1.96	1.97	1.85	1.86	0.02	0.321	**< 0.001**	0.992	2.08	2.07	2.04	1.99	0.04	0.412	0.144	0.652
FCR d 0–38	1.67	1.67	1.62	1.63	0.01	0.632	**< 0.001**	0.874	1.72	1.70	1.69	1.66	0.01	0.101	**0.026**	0.842

The table shows mean values. SEM: standard error of the means. ANOVA, Tukey’s range test, *n* = 8, α = 0.05. *P*-values are given for the main effects (crude protein (CP) and crude fat (CF)), separately for TN and HS conditions. As treatments were only applied during the finisher phase, *P-*values are only shown for this period. There were no interaction effects between CP and CF. Significant effects are highlighted in bold

*LowCP-ConF* Low crude protein with control fat, *LowCP-HighF* Low crude protein with high fat, *ConCP-HighF *Control crude protein with high fat, *ConCP-ConF* Control crude protein with control fat, *TN* Thermoneutral, *HS* heat stress, *BW* Body weight (g), *ADFI* Average daily feed intake (g/d), *ADG* Average daily gain (g/d), *FCR* Feed conversion ratio

Broilers fed the HighF diets showed a significantly lower ADFI (180.8 ± 6.85 g/d) during d 25–38 compared to the diets with control levels (186.7 ± 5.23 g/d) under TN conditions (*P* < 0.001). This effect was also observed considering the total rearing period (*P* = 0.021) and during HS conditions (*P* = 0.022). Under TN conditions, HighF resulted in reduced ADG (94.7 ± 3.90 g/d) compared to control levels (97.8 ± 3.81 g/d) (*P* = 0.010). In HS groups, ADG and FCR were not significantly different in HighF diets compared to control levels.

Mortality seemed numerically higher in broilers under HS conditions compared to TN groups. For example, the control group increased from 2.92% ± 1.17% to 9.17% ± 1.75% mortality. However, no statistical analysis could be performed on this data. There was more mortality in broilers fed HighF compared to control levels during HS conditions (*P* = 0.040; Table 3).

**Table 3:** Mortality during starter, grower and finisher with treatments applied during finisher under TN and HS conditions, %

Item	**TN**								**HS**							
	**LowCP-ConF**	**LowCP-HighF**	**ConCP-HighF**	**ConCP-ConF**	**SEM**	***P*-value**			**LowCP-ConF**	**LowCP-HighF**	**ConCP-HighF**	**ConCP-ConF**	**SEM**	***P*-value**		
						**CP × CF**	**CP**	**CF**						**CP × CF**	**CP**	**CF**
d 0–11	0.42	1.25	0.83	0.83	0.003				0.42	0.83	0.42	0.42	0.002			
d 11–25	2.08	0.83	1.67	1.67	0.004				3.35	0.83	2.51	2.92	0.005			
d 25–38	0.00	0.42	0.42	0.45	0.002	0.249	0.550	0.552	8.17	12.37	11.93	6.01	0.097	0.573	0.589	0.033
d 0–38	2.50	2.50	2.92	2.92	0.005	1.000	1.000	0.624	11.67	13.75	14.58	9.17	0.011	0.444	0.122	0.719

The table shows mean values. SEM: standard error of the means. Generalized linear model with binomial distribution, *n* = 8, α = 0.05. *P*-values are given for the main effects (crude protein (CP) and crude fat (CF)), separately for TN and HS conditions. As treatments were only applied during the finisher phase, *P-*values are only shown for this period. There were no interaction effects between CP and CF. Significant effects are highlighted in bold

*LowCP-ConF* Low crude protein with control fat, *LowCP-HighF* Low crude protein with high fat, *ConCP-HighF* Control crude protein with high fat, *ConCP-ConF* Control crude protein with control fat, *TN* Thermoneutral, *HS* Heat stress

### Breathing frequency, panting percentage, body temperature and lesions

**Fig. 2:**
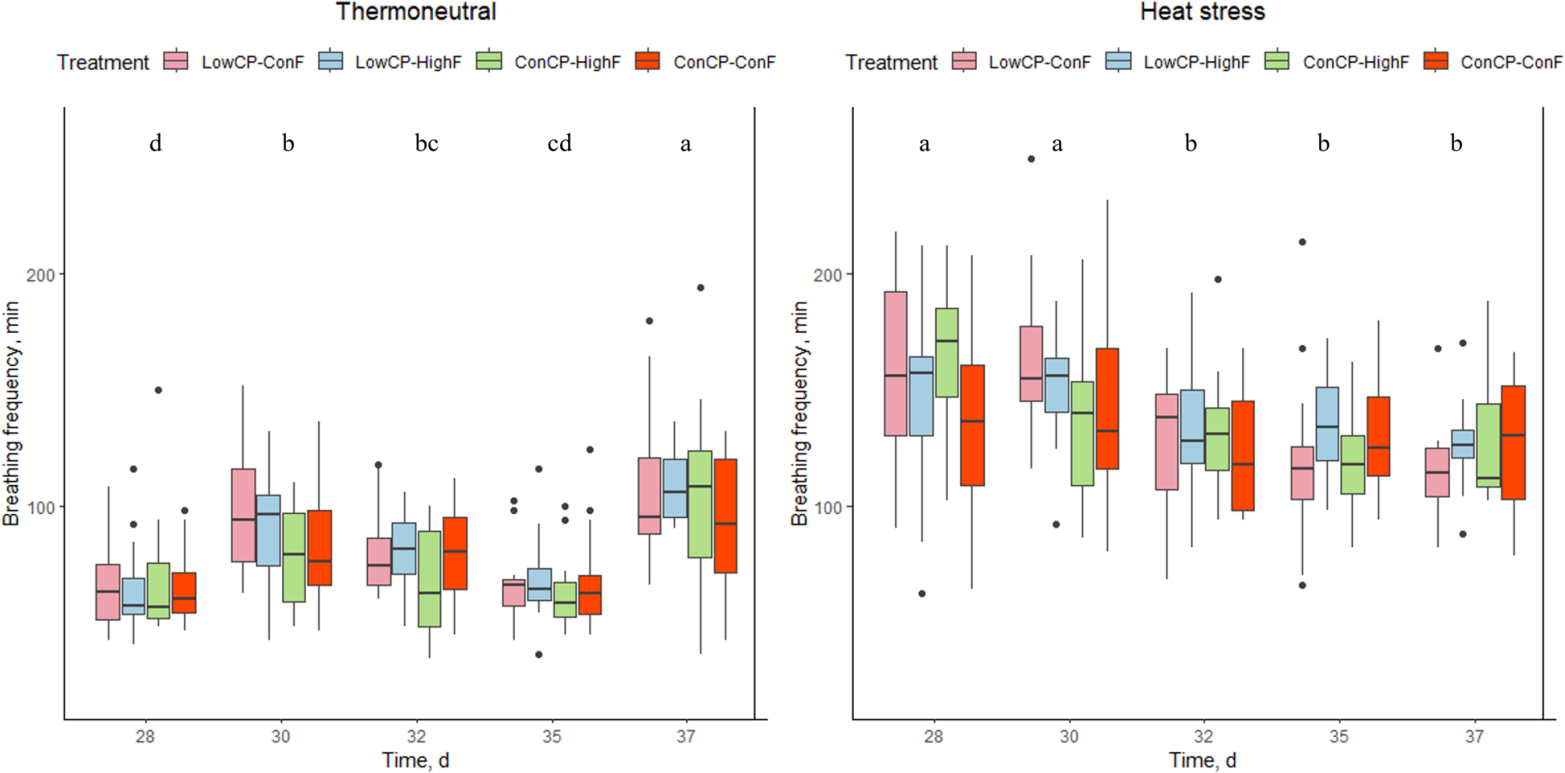
Breathing frequency (per min) of the broilers fed a low crude protein (LowCP-ConF), low crude protein and high crude fat (LowCP-HighF), high crude fat (ConCP-HighF) or a control (ConCP-ConF) diet under thermoneutral (TN) and heat stress (HS) conditions, determined on d 28, 30, 32, 35 and 37. ANOVA, Tukey’s range test, *n* = 8, α = 0.05. Statistical analysis was performed for TN and HS conditions separately. Different letters show statistically significant differences between measurement days

**Fig. 3:**
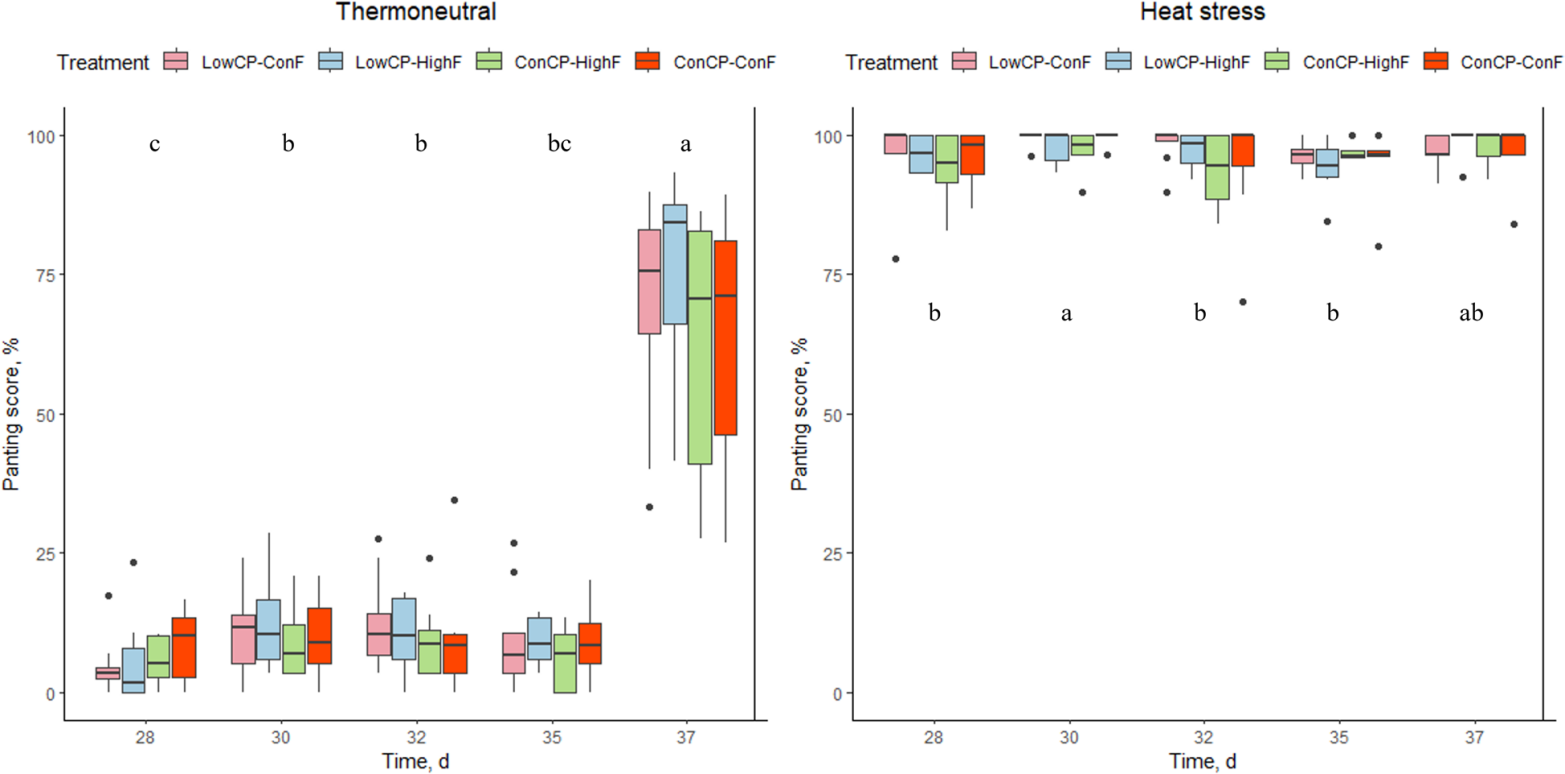
Panting percentage (%) of the broilers fed a low crude protein (LowCP-ConF), low crude protein and high crude fat (LowCP-HighF), high crude fat (ConCP-HighF) or a control (ConCP-ConF) diet under thermoneutral (TN) and heat stress (HS) conditions, determined on d 28, 30, 32, 35 and 37. ANOVA, Tukey’s range test, *n* = 8, α = 0.05. Statistical analysis was performed for TN and HS conditions separately. Different letters show statistically significant differences between measurement days

**Fig. 4:**
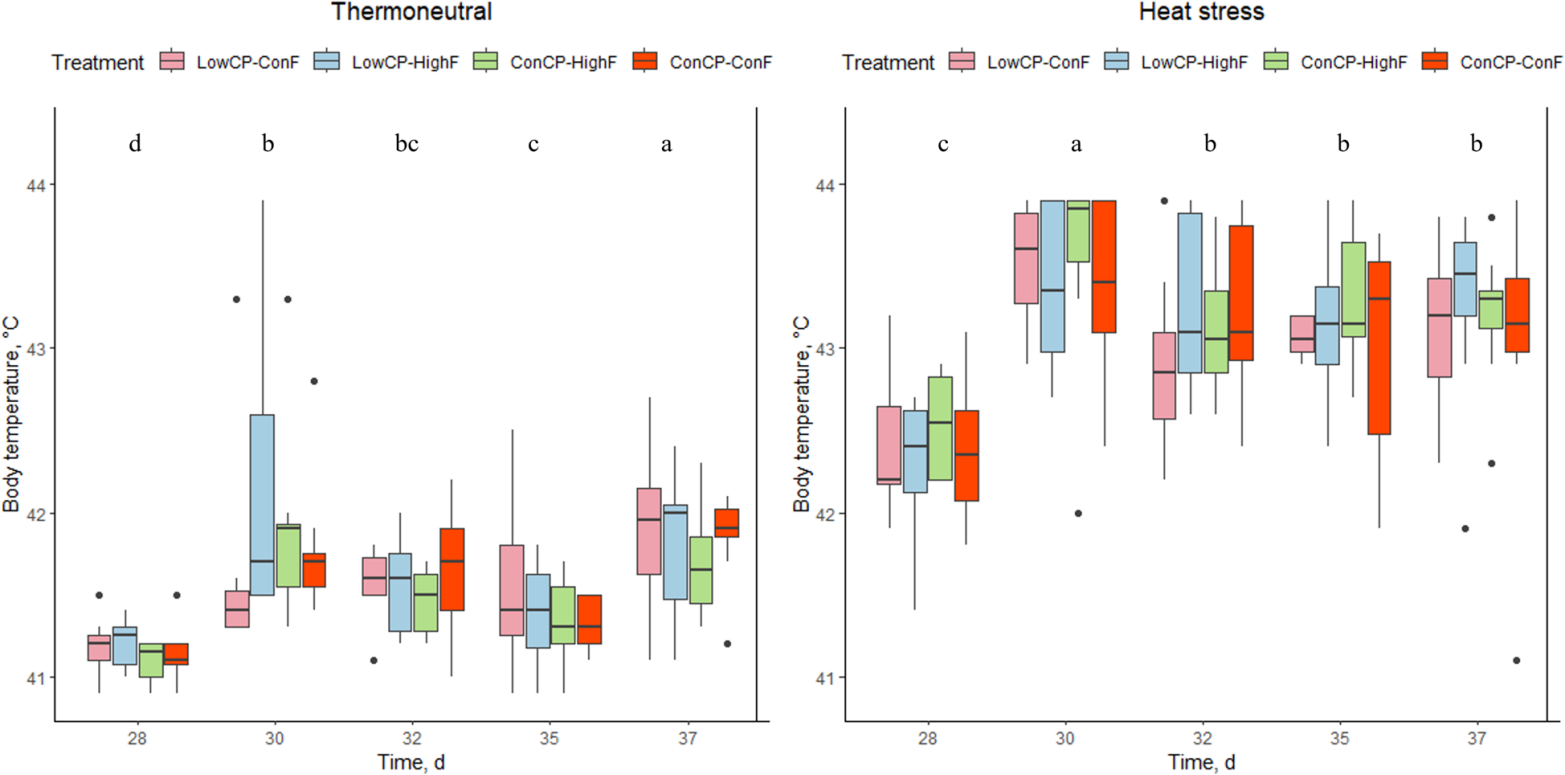
Body temperature (°C) of the broilers fed a low crude protein (LowCP-ConF), low crude protein and high crude fat (LowCP-HighF), high crude fat (ConCP-HighF) or a control (ConCP-ConF) diet under thermoneutral (TN) and heat stress (HS) conditions, determined on d 28, 30, 32, 35 and 37. ANOVA, Tukey’s range test, *n* = 8, α = 0.05. Statistical analysis was performed for TN and HS conditions separately. Different letters show statistically significant differences between measurement days

No significant interactions between crude protein and fat were found for breathing frequency, panting percentage, or body temperature, and these parameters were not significantly affected by the dietary treatments during either TN or HS conditions. Under HS conditions, a significant decrease in breathing frequency was observed starting from d 32 (Fig. 2). For the TN group, breathing frequency was significantly lower on d 28, compared to d 30, 32 and 37. Panting percentage in the HS group was significantly higher on d 30 compared to d 28, 32 and 35. For the TN group, a significant increase in panting was found for d 37 compared to the other measurement days (Fig. 3), because of increased stable temperature (max. 30.0 °C) following high environmental outdoor temperature (Fig. 1). Under HS conditions, body temperature was significantly higher on d 30 compared to the other measurement days. Furthermore, the body temperature of the broilers on d 28 was significantly lower compared to d 30, 32, 35 and 37. For the TN group, there was a significant increase in body temperature over the days. However, only body temperature on d 35 was lower compared to the previous measurement day (Fig. 4).

**Fig. 5:**
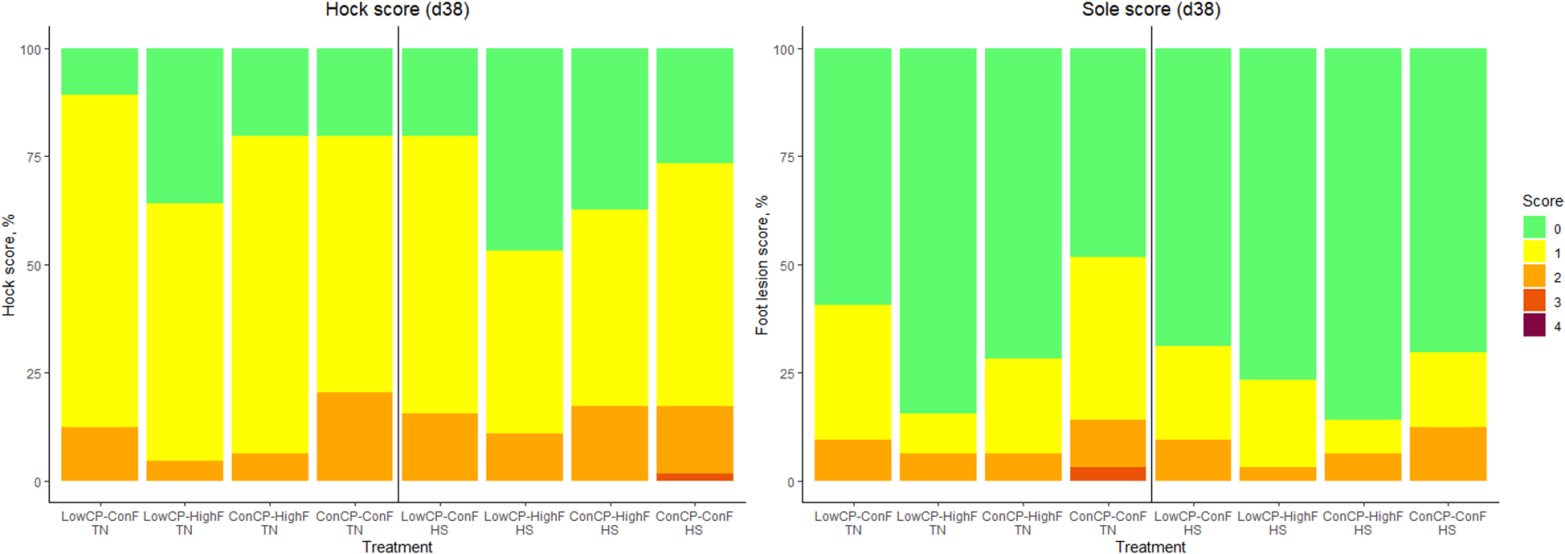
Hock lesion score (%) and foot lesion score (%) of the broilers fed a low crude protein (LowCP-ConF), low crude protein and high crude fat (LowCP-HighF), high crude fat (ConCP-HighF) or a control (ConCP-ConF) diet under thermoneutral (TN) and heat stress (HS) conditions, determined on d 38. Cumulative logit model, ANOVA, Tukey’s range test, *n* = 8, α = 0.05. Statistical analysis was performed for TN and HS conditions separately

There was a significant interaction of fat and crude protein on footpad lesions (d 38) during TN conditions (*P* < 0.001) and on hock lesions (d 38) during HS (*P* < 0.001) (Fig. 5). HighF diets had significantly lower hock lesion scores (d 38) during TN (*P* < 0.001). Footpad lesion scores were also significantly lower in HighF diets during HS conditions (*P* < 0.001). Crude protein level significantly affected hock lesions during TN (*P* < 0.001) and footpad lesions during HS (*P* < 0.001). However, post-hoc pairwise comparisons did not reveal significant differences between crude protein levels. Litter scores were not significantly affected.

### Physiological parameters

There were no interaction effects between crude protein and fat for the physiological parameters (Table 4). During both TN and HS conditions, there were no significant effects of the dietary treatments on BW, body temperature, abdominal fat pad mass, liver L*a*b* color space, liver mass, aspartate transaminase, creatine kinase, lactate and malondialdehyde levels in both plasma and liver tissue. Broilers fed a LowCP diet had a significantly lower uric acid level (4.75 ± 1.960 mg/dL; *P* = 0.007) and lower lactate dehydrogenase level (4,555.8 ± 1,233.15 IU/L; *P* = 0.035) compared to diets with control levels (5.98 ± 1.224 mg/dL and 5,521.1 ± 2,128.24 IU/L respectively) under TN conditions (*P* = 0.007). Under HS conditions, LowCP resulted in higher glucose levels (263.94 ± 30.389 mg/dL) compared to control levels (244.84 ± 30.627 mg/dL; *P* = 0.009).

**Table 4:** Effects of the different treatments during TN and HS conditions on physiological parameters at slaughter age

Item	**TN**								**HS**							
	**LowCP-ConF**	**LowCP-HighF**	**ConCP-HighF**	**ConCP-ConF**	**SEM**	***P*-value**			**LowCP-ConF**	**LowCP-HighF**	**ConCP-HighF**	**ConCP-ConF**	**SEM**	***P*-value**		
						**CP × CF**	**CP**	**CF**						**CP × CF**	**CP**	**CF**
Body temperature, °C	41.4	41.4	41.2	41.3	0.10	0.893	0.076	0.734	43.0	43.0	43.0	42.7	0.13	0.493	0.221	0.277
L* liver	31.3	32.5	29.9	29.7	1.42	0.742	0.168	0.643	28.0	28.4	29.0	26.4	4.54	0.560	0.794	0.438
a* liver	21.7	20.7	21.7	21.7	3.38	0.703	0.690	0.701	22.1	22.8	23.2	23.2	3.99	0.790	0.572	0.804
b* liver	19.9	19.3	18.8	18.6	2.47	0.678	0.349	0.863	18.5	20.2	19.8	18.5	1.41	0.847	0.809	0.140
Liver mass, g^1^	67.92	65.31	63.95	64.00	2.11	0.583	0.068	0.494	59.06	57.13	64.31	61.68	3.52	0.700	0.150	0.945
Abdominal fat mass, g^2^	39.96	37.87	38.45	35.32	2.12	0.189	0.143	0.646	38.11	42.68	39.31	37.20	2.38	0.432	0.238	0.162
MDA liver	153.0	145.0	143.8	169.0	11.4	0.374	0.454	0.073	151.6	151.7	139.7	150.9	12.3	0.473	0.461	0.465
Blood parameters																
Uric acid, mg/dL	4.66	4.85	5.53	6.44	0.41	0.188	**0.007**	0.389	4.78	4.35	5.48	4.71	0.50	0.242	0.300	0.739
AST, IU/L	393.33	390.40	416.25	500.11	34.3	0.252	0.068	0.222	412.84	486.32	392.99	443.41	37.8	0.116	0.422	0.766
CK, IU/L	28,454	24,455	27,739	35,679	3,789	0.584	0.150	0.104	23,654	22,812	21,071	21,618	2,495	0.951	0.425	0.769
LDH, IU/L	4,584.6	4,527.0	5,304.0	5,738.3	448.0	0.668	**0.035**	0.573	4,758.6	6,117.0	4,272.8	4,915.9	551	0.117	0.191	0.212
Lactate, mmol/L	9.79	8.15	9.25	8.39	0.74	0.091	0.819	0.613	7.87	6.34	7.42	7.08	0.70	0.176	0.838	0.393
Glucose, mg/dL	232.3	242.2	242.3	231.8	8.78	0.969	0.979	0.238	262.5	265.3	241.6	248.1	10.3	0.450	**0.009**	0.810
MDA plasma	11.47	12.18	14.48	12.50	1.38	0.516	0.093	0.170	12.05	17.0	16.0	15.3	2.30	0.162	0.448	0.066

The table shows mean values. SEM: standard error of the means. ANOVA, Tukey’s range test, *n* = 8, α = 0.05. *P*-values are given for the main effects (crude protein (CP) and crude fat (CF)), separately for TN and HS conditions. There were no interaction effects between CP and CF. Significant effects are highlighted in bold

*LowCP-ConF* Low crude protein with control fat, *LowCP-HighF* Low crude protein with high fat, *ConCP-HighF* Control crude protein with high fat, *ConCP-ConF* Control crude protein with control fat, *TN* Thermoneutral, *HS* Heat stress, *BW* Body weight (g), *MDA* Malondialdehyde (nmol MDA equivalents/g (liver) or mL (plasma) sample), L* Lightness of the liver, a* Green-red opponent of the liver, b* Blue-yellow opponent of the liver, *AST* Aspartate aminotransferase, *CK* Creatine kinase, *LDH* Lactate dehydrogenase

^1^Absolute weight was used with bodyweight as covariable. *P*-value for body weight (BW) in TN group was *P* = 0.003, and for the HS group: *P* = 0.002

^2^Absolute weight was used with bodyweight as covariable. *P*-value for body weight (BW) in TN group was *P* = 0.007, and for the HS group: *P* = 0.223

### Slaughter yield and meat quality

There were no interaction effects between crude protein and fat, nor significant effect of dietary treatments, for meat quality parameters during both TN and HS conditions (Table 5). Furthermore, there were no significant effects on shear force, thawing loss or cooking loss. Broilers fed LowCP had a significantly higher breast meat pH (6.38 ± 0.121) compared to control CP levels (6.32 ± 0.118) under HS conditions (*P* = 0.035). Additionally, LowCP significantly increased breast meat redness (a*) during both TN (*P* = 0.023) and HS (*P* = 0.029) conditions, but did not affect L* and b* values.

**Table 5:** Effects of the different treatments during TN and HS conditions on slaughter yield and meat quality at slaughter age

Item	**TN**								**HS**							
	**LowCP-ConF**	**LowCP-HighF**	**ConCP-HighF**	**ConCP-ConF**	**SEM**	***P*-value**			**LowCP-ConF**	**LowCP-HighF**	**ConCP-HighF**	**ConCP-ConF**	**SEM**	***P*-value**		
						**CP × CF**	**CP**	**CF**						**CP × CF**	**CP**	**CF**
Carcass yield, %	65.22	64.58	64.82	64.86	0.36	0.344	0.366	0.321	66.04	66.36	67.18	66.77	0.45	0.925	0.093	0.417
Breast yield, %	30.27	29.51	30.13	29.91	0.49	0.293	0.750	0.506	28.49	28.25	29.01	28.65	0.49	0.539	0.343	0.882
Drumstick yield, %	13.85	14.09	14.13	14.13	0.19	0.597	0.678	0.169	14.29	14.46	14.69	14.76	0.29	0.613	0.568	0.608
Thigh yield, %	27.62	28.07	27.85	27.64	0.28	0.504	0.383	0.511	28.42	28.75	28.40	28.40	0.32	0.654	0.187	0.847
Wing yield, %	10.51	10.47	10.41	10.48	0.14	0.892	0.725	0.639	10.76	10.34	10.55	10.59	0.16	0.195	0.930	0.120
Breast meat color																
L*	55.1	54.5	55.1	55.2	0.51	0.597	0.427	0.504	53.6	54.1	54.4	55.2	0.48	0.155	0.054	0.821
a*	6.1	6.4	5.9	5.7	0.26	0.890	**0.023**	0.200	6.2	6.2	5.7	5.7	0.23	0.989	**0.029**	0.924
b*	13.4	13.1	13.1	13.0	0.22	0.459	0.265	0.822	12.6	13.0	13.0	12.8	0.21	0.753	0.622	0.138
Meat quality																
pH	6.25	6.26	6.25	6.24	0.02	0.880	0.449	0.347	6.39	6.36	6.30	6.35	0.02	0.912	**0.035**	0.120
Shear force, N	7.99	7.92	7.92	7.89	0.42	0.909	0.909	0.941	8.42	8.29	7.61	8.53	0.43	0.326	0.499	0.197
Thawing loss, %	6.41	6.65	5.83	5.59	0.43	0.990	0.069	0.592	7.04	6.15	6.88	7.22	0.44	0.529	0.303	0.167
Cooking loss (fresh), %	23.59	24.85	23.23	23.74	0.74	0.230	0.334	0.567	24.72	24.18	25.35	24.56	0.80	0.412	0.534	0.877
Cooking loss (thawed), %	18.35	19.51	18.50	19.22	0.67	0.157	0.897	0.688	19.03	19.27	19.85	18.77	0.64	0.494	0.795	0.291
Crude fat in breast meat, %^1^	7.94	9.58	7.08	9.23	1.06	0.087	0.589	0.818	8.30	6.95	6.77	8.76	0.71	0.633	0.831	**0.017**
Crude protein in breast meat, %^1^	90.2	88.9	90.2	87.8	1.04	0.091	0.652	0.616	89.8	90.1	91.8	89.1	0.79	0.101	0.506	**0.047**

The table shows mean values. SEM: standard error of the means. ANOVA, Tukey’s range test, *n* = 8, α = 0.05. *P*-values are given for the main effects (crude protein (CP) and crude fat (CF)), separately for TN and HS conditions. There were no interaction effects between CP and CF. Significant effects are highlighted in bold

*LowCP-ConF* Low crude protein with control fat, *LowCP-HighF* Low crude protein with high fat, *ConCP-HighF* Control crude protein with high fat, *ConCP-ConF* Control crude protein with control fat, *TN* Thermoneutral, *HS* Heat stress, L* Lightness, a* Greenred opponent, b* Blue-yellow opponent

^1^Dry matter basis

HighF only affected breast meat composition. Conversely, broilers HighF had a significantly lower fat percentage (6.86 ± 1.559%; *P* = 0.017) and higher protein percentage (90.95% ± 1.686%; *P* = 0.047) in breast meat compared to the diets with control levels (8.53% ± 2.286% and 89.42% ± 2.724% respectively). These effects were only observed during HS conditions.

**Fig. 6:**
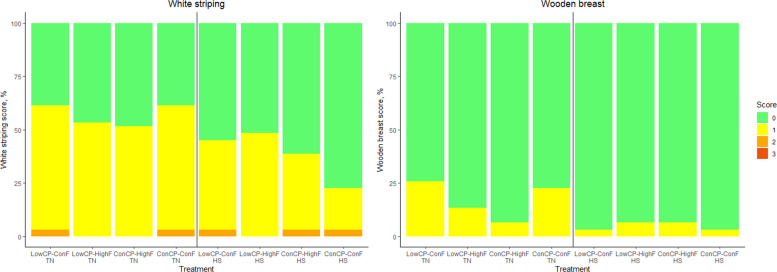
White striping score (%) and wooden breast score (%) of the broilers fed a low crude protein (LowCP-ConF), low crude protein and high crude fat (LowCP-HighF), high crude fat (ConCP-HighF) or a control (ConCP-ConF) diet under thermoneutral (TN) and heat stress (HS) conditions, determined on d 38. Cumulative logit model, ANOVA, Tukey’s range test, *n* = 8, α = 0.05. Statistical analysis was performed for TN and HS conditions separately

White striping (WS) scores were affected by fat level during TN conditions (*P* < 0.001) and HS conditions (*P* < 0.001). Crude protein levels significantly affected WS scores (*P* = 0.004) during HS conditions. However, post-hoc pairwise comparisons did not reveal significant differences between individual fat or protein levels. Wooden breast (WB) scores were significantly affected by crude protein level during TN and HS (*P* < 0.001), but post-hoc pairwise comparisons did not reveal significant differences between individual crude protein levels (Fig. 6). There were no effects on spaghetti meat as nearly all scores were zero.

## Discussion

In absence of an interaction between dietary factors, fat and crude protein, the two main effects had an impact on performance in both climate conditions.

It was noted that LowCP diets negatively impacted broiler performance under thermoneutral conditions, but the effect remained absent when heat stress was applied. Under TN, LowCP increased ADFI, which may imply that potential amino acid deficiencies influenced feed intake, impairing the ideal protein concept, although other factors may be involved [21, 24, 42]. Nevertheless, feed intake is influenced by other factors, e.g., protein source and quality [17]. In LowCP diets, the reduction in CP was achieved by replacing soybean meal with the supplementation of crystalline amino acids to reach a similar level of SID lysine and maintaining the amino acid profile, which may have contributed to this effect. Despite the increased feed intake, broilers on LowCP diets still exhibited reduced ADG, resulting in a less efficient feed conversion. These findings are in accordance with previous studies and may be related to a higher digestive heat production linked to increased ADFI [16, 43]. Notably, live slaughter weights were not significantly affected by crude protein level. During HS, no effects of CP level on performance were observed, possibly due to the inherently reduced metabolic rate under HS conditions, which lowers the overall protein requirement [7, 44].

Nonetheless, performance improvements typically associated with the other main factor in the study, high-fat supplementation, were not observed. HighF diets reduced ADFI in both TN and HS conditions, which contradicts previous studies reporting increased ADFI presumably due to the enhanced palatability of high-fat diets [7, 25]. A possible hypothesis could be the slight increase in the level of metabolizable energy when adding fat (i.e., 2,919 kcal/kg and 3,005 kcal/kg AME_n_ on average for ConF and HighF, respectively), which could elicit a compensatory decrease in feed intake to maintain energy intake. Additionally, an altered pellet quality due to the higher added fat could also be a factor to consider. This altered feed intake would influence the absolute protein intake, consequently ADG was reduced under TN conditions, although these negative trends on growth and FCR observed under TN conditions were not observed during HS conditions, supporting previous observations that broilers might have different energy and protein requirements under HS compared to TN conditions [8].

Feeding LowCP diets under TN conditions significantly influenced metabolic responses. Plasma uric acid concentration was reduced, in line with findings in literature [14, 24, 27]. This decline may result from a lower absolute protein intake, but could also indicate reduced protein catabolism as a possible adaptive response to amino acid deficiency, countering the use of excess amino acids as energy source and repurposing amino acids in non-essential amino acids rather that excreting them [24, 27]. Plasma AST and CK as markers for respectively. liver damage and muscle damage showed no differences across treatments and climate conditions. However, similar to uric acid, lactate dehydrogenase activity was decreased in broilers receiving LowCP diets under TN conditions, which may reflect a limited amino acid availability for anaerobic glycolysis [45]. Although, this was not reflected in lactate levels. Additionally, the relative energy surplus in these diets reduces the reliance on gluconeogenesis which may contribute to the downregulation of lactate dehydrogenase [45, 46]. As observed in performance, these metabolic shifts were also absent under HS, which might be due to HS adversely affecting energy:protein metabolism, blunting potential dietary effects [47, 48]. Plasma glucose levels were unaffected under TN, despite a higher energy intake per unit of metabolic BW in broilers receiving a dietary treatment (higher energy-to-protein ratio). In contrast, during HS, broilers on LowCP diets, showed elevated plasma glucose, which could find its origin in the excess energy available relative to crude protein during heat stress causing less energy dissipation trough heat, eliciting insulin resistance.

Despite the minor metabolic shifts in HS groups, mortality was higher in chickens fed HighF diets. A potential explanation could be the oxidation of fat during feeding or digestion, as polyunsaturated fats are particularly sensitive to oxidation [28]. However, only a trend for higher MDA levels in plasma but not in liver tissue was observed in this experiment.

The altered fat and crude protein levels did not affect other physical responses in the broilers despite the hypothesis that LowCP during HS would reduce breathing frequency, panting percentage and body temperature, due to the high heat increment associated with CP [9, 13]. Similarly, the inclusion of extra fat had no effect on these parameters either, whereas Attia et al. [44] reported decreased body temperature and respiration rate in broilers receiving a high-energy diet (3,360 kcal, 14.06 MJ/kg) supplemented with fat and a higher CP level (22%).

Carcass yield and breast muscle composition were largely unaffected by dietary crude protein and fat modifications, despite differences in performance. Although low-protein diets generally impair growth performance, they do not necessarily compromise carcass yield or part weights [49–51]. However, yield and relative parts are expressed as percentage, and organ fat and fat pad are removed during slaughter explaining that whilst growth may be affected, relative lean carcass composition may be unaffected. In contrast, Zarate et al. [52] observed reduced carcass and part yields in fast-growing broilers receiving similar CP levels (17%) under HS. Other studies also demonstrated that carcass yield and breast weight may increase linearly with higher protein levels [26]. Ghazalah et al. [25] observed improved performance with high fat diets, yet carcass traits remain unaffected, as observed in this study. All dietary treatments had a higher energy-to-protein ratio compared to the control, which could promote fat deposition [27, 46]. However, no increase in fat deposition in breast meat was detected. On the contrary, diets with HighF were observed to have less fat deposition and higher protein levels in breast muscles of HS broilers, suggesting that these diets may better support lean muscle development under HS conditions [27]. Although reduced CP intake is typically associated with lower breast meat protein content, this was not observed, which is in accordance with Furlan et al. [17]. Lower CP increased breast meat redness, which might be related to slower growth rates, allowing more extensive vascular development in the breast muscle [24, 53].

The absence of interaction effects between crude protein and fat suggests that their individual effects may have a more prominent role under the tested conditions. Alternatively, the range or balance of dietary ratios applied may not have been optimal to elicit interactions, or metabolic ceilings may have limited dietary interactions, especially during HS. Moreover, modifying nutrient levels by altering feed composition inevitably affects ingredient ratios and matrix complexity, which could have influenced the outcomes as well.

## Conclusion

Under TN conditions, a reduction in CP from 18.1%–18.7% to 17.0%–17.2% increased feed intake but impaired growth performance and feed efficiency, without affecting carcass yield. Under HS, these negative effects were not observed, rendering these LowCP diets neither clearly beneficial nor harmful during HS. Conversely, increasing CF from 5.9%–6.3% to 7.9%–8.0% reduced feed intake under both TN and HS, likely due to the higher energy content of the diet. While it impaired growth performance under TN, and similar to LowCP, no such effects were seen under HS. This implies that protein and energy requirements and ratios are different depending on the environmental conditions. The HighF diet improved breast meat composition under HS, indicating potential benefits for meat quality. However, neither LowCP nor HighF improved HS tolerance based on body temperature, panting, or respiration rate. HighF requires caution due to its negative impact on survival during heat exposure. Future research should prioritize understanding amino acid requirements and energy-to-protein ratios under HS conditions, especially to uncover the effect on energy homeostasis.

## Data Availability

The datasets used and/or analysed during the current study are available from the corresponding author on reasonable request.

## Abbreviations

ADFI: Average daily feed intake
ADG: Average daily growth
AST: Aspartate transaminase
BW: Body weight
CF: Crude fat
CK: Creatine kinase
CP: Crude protein
FCR: Feed conversion ratio
GE: Gross energy
HS: Heat stress
LDH: Lactate dehydrogenase
MDA: Malondialdehyde
ME: Metabolizable energy
NE: Net energy
RH: Relative humidity
SID: Standardized ileal digestibility
Tb: Body temperature
THI: Temperature-humidity index
TN: Thermoneutral
WB: Wooden breast
WS: White striping
